# Performance of the quick Sequential (sepsis-related) Organ Failure Assessment score as a prognostic tool in infected patients outside the intensive care unit: a systematic review and meta-analysis

**DOI:** 10.1186/s13054-018-1952-x

**Published:** 2018-02-06

**Authors:** Jae-Uk Song, Cheol Kyung Sin, Hye Kyeong Park, Sung Ryul Shim, Jonghoo Lee

**Affiliations:** 1 Division of Pulmonary and Critical Care Medicine, Department of Internal Medicine, Kangbuk Samsung Hospital, Sungkyunkwan University School of Medicine, Seoul, South Korea; 2 Department of Internal Medicine, Samsung Changwon Hospital, Sungkyunkwan University School of Medicine, Changwon, South Korea; 3 Division of Pulmonary and Critical Care Medicine, Department of Internal Medicine, Ilsan Paik hospital, Inje University College of Medicine, Goyang-si, South Korea; 4 Institute for Clinical Molecular Biology Research, Soonchunhyang University Hospital, Soonchunhyang University College of Medicine, Seoul, South Korea; 5 Department of Internal Medicine, Jeju National University Hospital, Jeju National University School of Medicine, Aran 13 gil 15, Jeju-si, Jeju Special Self-Governing Province 63241 South Korea

**Keywords:** qSOFA, Mortality, Sepsis, Intensive care unit, Emergency department

## Abstract

**Background:**

The usefulness of the quick Sequential (Sepsis-related) Organ Failure Assessment (qSOFA) score in providing bedside criteria for early prediction of poor outcomes in patients with suspected infection remains controversial. We investigated the prognostic performance of a positive qSOFA score outside the intensive care unit (ICU) compared with positive systemic inflammatory response syndrome (SIRS) criteria.

**Methods:**

A systematic literature search was performed using MEDLINE, Embase, and the Cochrane Central Register of Controlled Trials. Data were pooled on the basis of sensitivity, specificity, and diagnostic OR. Overall test performance was summarized using a hierarchical summary ROC and the AUC. Meta-regression analysis was used to identify potential sources of bias.

**Results:**

We identified 23 studies with a total of 146,551 patients. When predicting in-hospital mortality in our meta-analysis, we identified pooled sensitivities of 0.51 for a positive qSOFA score and 0.86 for positive SIRS criteria, as well as pooled specificities of 0.83 for a positive qSOFA score and 0.29 for positive SIRS criteria. Discrimination for in-hospital mortality had similar AUCs between the two tools (0.74 vs. 0.71; *P* = 0.816). Using meta-regression analysis, an overall mortality rate ≥ 10% and timing of qSOFA score measurement could be significant sources of heterogeneity. For predicting acute organ dysfunction, although the AUC for a positive qSOFA score was higher than that for positive SIRS criteria (0.87 vs. 0.76; *P* < 0.001), the pooled sensitivity of positive qSOFA score was very low (0.47). In addition, a positive qSOFA score tended to be inferior to positive SIRS criteria in predicting ICU admission (0.63 vs. 0.78; *P* = 0.121).

**Conclusions:**

A positive qSOFA score had high specificity outside the ICU in early detection of in-hospital mortality, acute organ dysfunction, and ICU admission, but low sensitivity may have limitations as a predictive tool for adverse outcomes. Because between-study heterogeneity was highly represented among the studies, our results should be interpreted with caution.

**Electronic supplementary material:**

The online version of this article (10.1186/s13054-018-1952-x) contains supplementary material, which is available to authorized users.

## Background

Sepsis is defined as life-threatening organ dysfunction that is caused by a dysregulated host response to infection [1]. It is a common cause of admission to the intensive care unit (ICU) and can lead to multiple organ dysfunction syndrome and death [2]. It is essential to differentiate sepsis from an uncomplicated infection because sepsis is associated with poorer outcomes [3]. Early recognition of sepsis can improve outcomes of these patients through corresponding interventions, which include adequately administering fluids and appropriate antibiotics [3]. However, because sepsis is a complex, heterogeneous disease, it is often difficult for clinicians to promptly identify patients with sepsis.

There are no gold standard tests or diagnostic criteria to detect patients with sepsis. For more than two decades, the systemic inflammatory response syndrome (SIRS) criteria have been used in the diagnosis of sepsis [4, 5]. Researchers in several studies have reported controversies regarding the applicability of SIRS, and the SIRS criteria have also been criticized as a sepsis screening tool because of inadequate specificity and sensitivity [4, 5]. In 2016, the Society of Critical Care Medicine (SCCM)/European Society of Intensive Care Med (ESICM) task force released the Third International Consensus Definitions for Sepsis and Septic Shock (Sepsis-3) as a new definition for sepsis [1]. The consensus definition replaced the SIRS criteria with the Sequential (Sepsis-related) Organ Failure Assessment (SOFA) score [1]. In addition, the quick SOFA (qSOFA) score was introduced as a bedside criterion to facilitate the identification of patients with suspected infection who are likely to have poor outcomes [1]. Data published shortly after the establishment of Sepsis-3 demonstrated that the predictive validity of qSOFA for in-hospital mortality was statistically greater than either the original SOFA or SIRS criteria in encounters with suspected infection outside the ICU [6]. Researchers in several studies have examined the predictive performance of the qSOFA score for in-hospital mortality in these patients. These studies have generated conflicting evidence, and it is currently unclear whether the qSOFA score has prognostic value for unfavorable outcomes in patients with a suspected infection.

There have been no published meta-analyses of the predictive performance of the qSOFA score. The aim of the present study was to evaluate the prognostic value of a positive qSOFA score compared with positive SIRS criteria for early identification of in-hospital mortality in patients with suspected infection outside the ICU. We also compared the discriminatory capacity between positive qSOFA score and positive SIRS criteria in predicting acute organ dysfunction and ICU admission.

## Methods

### Data sources and search strategy

This meta-analysis is reported in accordance with the Preferred Reporting Items for Systematic reviews and Meta-Analyses statement [7]. The study protocol was registered with the PROSPERO International Prospective Register of Systematic Reviews (http://www.crd.york.ac.uk/PROSPERO/display_record.asp?ID=CRD42017074766). To identify potentially relevant articles, we conducted a comprehensive search of three electronic databases (MEDLINE, Embase, and the Cochrane Central Register of Controlled Trials) up to July 1, 2017. We also performed a manual search of the references listed in relevant review articles. The detailed study protocol and search strategies are provided in Additional file 1. Because this study was a systematic review of published articles, neither informed consent nor ethics approval was required.

### Inclusion criteria

We included a study in our analysis if it met the following criteria:

1. The study targeted patients with suspected or confirmed infection outside the ICU.
2. The study evaluated the qSOFA score as a predictive tool for predicting in-hospital mortality, acute organ dysfunction, or ICU admission.
3. The study provided sufficient data to calculate absolute numbers of true-positive, false-positive, false-negative, and true-negative results.

Studies published as full-length articles or letters in peer-reviewed English-language journals were eligible.

### Data extraction, definitions, and outcomes

JUS and JL independently extracted potentially relevant studies and reviewed each study according to the predefined criteria for eligibility. We extracted data from the selected studies. Any disagreement in the process of study selection or data extraction was resolved by discussion. A predefined form was used to extract data from each study. Extracted information included details of patient demographics, the study design, and objectives.

The qSOFA score consists of three clinical variables: altered mentation, systolic blood pressure < 100 mmHg, and respiratory rate > 22 breaths/minute [1]. The score ranges from 0 to 3, and a positive qSOFA score is defined as 2 or more points [1]. The SIRS criteria were defined as a respiratory rate > 20 breaths/minute or partial pressure of carbon dioxide < 32 mmHg, a body temperature > 38 °C or < 36 °C, a heart rate > 90 beats/minute, and a white blood cell count > 12,000/mm^3^ or < 4000/mm^3^, or > 10% bands [8]. A positive SIRS criterion was also defined as ≥ 2 points [8]. Acute organ dysfunction was defined as an acute 2-point increase in the SOFA score following the proposed Sepsis-3 definitions [1]. In case of studies using the Sepsis-2 definitions, the development of severe sepsis (two or more SIRS signs plus one additional sign of organ failure) was considered as acute organ dysfunction [8, 9]. Outside the ICU included out-of-hospital, emergency department (ED), or general hospital ward settings.

The primary outcome was in-hospital mortality. For trials in which researchers did not investigate in-hospital mortality, we used the 28- or, 30-day mortality instead [10]. In addition, we extracted the data for SIRS from the published materials for qSOFA, and a positive qSOFA score was used to assess the prognostic performance by comparing it with positive SIRS criteria. The secondary outcome was acute organ dysfunction and ICU admission.

### Quality assessment

As recommended by the Cochrane Collaboration, we used the Quality Assessment of Diagnostic Accuracy Studies (QUADAS)-2 tool to assess the risk of bias in diagnostic test accuracy [11]. A detailed quality assessment is provided in Additional file 1.

### Data synthesis and statistical analysis

The data were presented as mean values for continuous variables and as frequencies (percent) for categorical variables. For diagnostic meta-analysis, we extracted the number of patients with a true-positive, false-positive, false-negative, and true-negative test result either directly or through a recalculation that was based on the reported measures of accuracy in combination with the prevalence and sample size in the included study. We calculated the pooled sensitivity and specificity, positive likelihood ratio (PLR), negative likelihood ratio (NLR), diagnostic OR (DOR), and AUC as point estimates with 95% CI [12]. We also constructed hierarchical summary ROC (HSROC) curves to overcome some limitations of the traditional summary ROC curve procedure [13], which was closely related to a bivariate random effects meta-analysis [13]. Between-study statistical heterogeneity was assessed using the*I*^2^ statistic and Cochran’s *Q* test [14]. Heterogeneity was assessed using *I*^2^ statistics on a scale of 0–100%. If *I*^2^ was > 50%, a random-effects model was used; otherwise, a fixed-effects model was used [14]. An *I*^2^ > 50% indicated a substantial level of between-study heterogeneity. In cases of substantial heterogeneity, analysis via meta-regression was performed to identify potential sources of bias [15]. If potential sources were found, additional meta-regression was conducted using a generalization of Moses-Littenberg linear models. The model was weighted by the inverse of the variance or study size [16]. Publication bias was evaluated using the Deek test for funnel plot asymmetry [17]. A *P* value < 0.05 was considered statistically significant. All analyses were performed using Meta-DiSc software (version 1.4; http://www.hrc.es/investigacion/metadisc_en.htm) and Stata statistical software (version 14.2; StataCorp LP, College Station, TX, USA).

## Results

### Study search

**Fig. 1:**
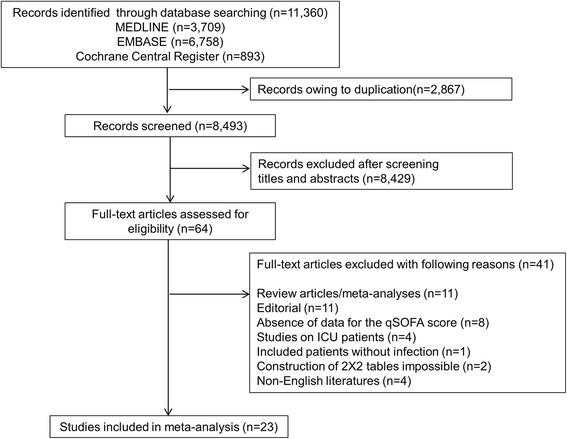
Flow diagram for the identification of eligible studies. *ICU* Intensive care unit, *qSOFA* Quick Sequential (Sepsis-related) Organ Failure Assessment

The flowchart in Fig. 1 shows the literature search process. A total of 11,360 published articles were initially identified (3709 articles from MEDLINE, 6758 articles from Embase, and 893 articles from the Cochrane Library). After removing duplicate articles, we screened 8493 potentially eligible articles. Of these articles, 8430 were excluded on the basis of title and abstract. A total of 63 articles underwent full-text review. Forty-one articles were excluded for the reasons presented in Fig. 1. Finally, a total of 23 articles met our inclusion criteria [6, 18–39]. All studies were published between 2016 and 2017. Features of the included studies are shown in Table 1. The number of patients in each trial ranged from 151 to 66,522, and the overall mortality rate in each study ranged from 2.8% to 33.0%. Researchers in 20 studies reported the discriminatory capacity of the qSOFA score for predicting mortality [6, 18–22, 24–32, 34, 36–39]. In 11 studies, investigators compared the accuracy of positive qSOFA score and positive SIRS criteria for predicting mortality [6, 18, 19, 21, 22, 24–26, 28, 37, 39]. The QUADAS assessment is summarized in Additional file 2. Overall, the quality of the studies was deemed satisfactory. However, the QUADAS tool showed that unclear blinding during interpretation of results and lack of reporting for uninterpretable results may be potential sources of bias. Withdrawals from some studies were not clearly explained, which could also have resulted in bias. The funnel plots and regression tests indicated no significant publication bias (*see* Additional file 3).

**Table 1:** Characteristics of studies included in the meta-analysis

Author, year [reference]	Design	Country	Total no. of patients	Mean age, years	Male sex (%)	Location	Overall mortality rate (%)	qSOFA score recorded	Measured mortality	Participant selection	Primary study aim
April et al., 2017 [18]	Retrospective, single-center, cohort study	USA	214	68	59	ED	18.2	Worst values during ED stay	In-hospital mortality	Suspected infection, admitted to ICU	Comparison of prognostic accuracy of qSOFA and SIRS for predicting in-hospital mortality
Askim et al., 2017 [19]	Prospective, single-center, observational study	Norway	1535	62	53	ED	4.4	ED arrival	7- and 30-day mortality	Suspected infection	Clinical usefulness of qSOFA to predict severe sepsis and 7- and 30-day mortality
Chen et al., 2016 [20]	Retrospective, single-center, observational study	China	1631	73	59	ED	33	ED arrival	28-day mortality	Community-acquired pneumonia	Comparison of prognostic performance of qSOFA, CRB-65, and CRB
Churpek et al., 2017 [21]	Retrospective, single-center, observational study	USA	30,677	58	47	ED, ward	5.4	At time of initial suspicion of infection	In-hospital mortality	Suspected infection	Comparison of qSOFA with other commonly used early warning scores for in-hospital mortality
Donnelly et al., 2017 [22]	Retrospective, multicenter, cohort study	USA	2593	67	40	NA	11.3	Worst values within 28 h of hospital admission	28-day and 1-year mortality	Suspected infection	Incidence and long-term outcomes of patients diagnosed with sepsis and septic shock
Dorsett et al., 2017 [23]	Retrospective, single-center, observational study	USA	152	NA	NA	ED	NA	Prehospital, upon ED arrival, and during ED stay	NA	Suspected infection	Prehospital qSOFA score in early identification of patients with severe sepsis or septic shock
Finkelsztein et al., 2017 [24]	Prospective, single-center, cohort study	USA	151	64	55	ED, ward	19	Within 8 h before ICU admission	In-hospital mortality	Suspected infection, admitted to medical ICU	Comparison of discriminatory capacity of qSOFA vs. SIRS criteria for predicting in-hospital mortality and ICU-free days
Forward et al., 2017 [25]	Retrospective, single-center, observational study	Australia	162	NA	NA	Non-ICU	15.5	Within 24 h of deterioration	In-hospital mortality	Suspected infection	Comparison of prognostic performance of qSOFA, SIRS, and SK criteria
Freund et al., 2017 [26]	Prospective, multicenter, cohort study	Europe	879	67	53	ED	8.4	Worst values during ED stay	In-hospital mortality	Suspected infection	Validation of qSOFA as mortality predictor comparing SIRS with SOFA
Giamarellos-Bourboulis et al., 2017 [27]	Retrospective, multicenter, cohort study	Greece	3436	NA	NA	ED, ward	25.2	Initial values measured during admission to ED	In-hospital mortality	Suspected or confirmed infection	Sensitivity of qSOFA for early assessment of mortality and organ dysfunction
Henning et al., 2017 [28]	Post hoc analysis	USA	7637	58	50	ED	14.2	Worst values during ED stay	In-hospital mortality	Suspected infection	Performance of qSOFA predicting in-hospital mortality
Huson et al., 2017 [29]	Retrospective, single-center, observational study	Gabon	329	34	38	Non-ICU	4.5	At time of initial suspicion of infection	In-hospital mortality	Suspected infection	Predictive value of qSOFA score for mortality
Hwang et al., 2017 [30]	Retrospective, single-center, cohort study	South Korea	1395	65	56	ED	15	ED arrival and within 3, 6, and 24 h	In-hospital and 28-day mortality	Severe sepsis or septic shock	Diagnostic performance of positive qSOFA score for predicting 28-day mortality among critically ill patients with sepsis
Kim et al., 2017 [31]	Retrospective, single-center, observational study	South Korea	615	54	33	Non-ICU	3.2	At time of initial suspicion of infection	28-day mortality	Neutropenic fever	Predictive performance of qSOFA as screening tool for sepsis, mortality, and ICU admission
Kolditz et al., 2017 [32]	Retrospective, multicenter, observational study	Germany	9327	64	56	Non-ICU	3.0	At time of initial suspicion of infection	30-day mortality	Community-acquired pneumonia	Comparison of qSOFA and CRB-65 for risk prediction
Mellhammar et al., 2017 [33]	Retrospective population-based study	Sweden	339	NA	NA	Non-ICU	NA	Within ± 12 h from initiation of antibiotic therapy	NA	Suspected infection	Incidence of sepsis with organ dysfunction
Park et al., 2017 [34]	Retrospective, single-center, observational study	South Korea	1009	67	45	ED	15.8	ED arrival	In-hospital mortality	Suspected infection	Comparison of performance of qSOFA and SIRS to predict development of organ failure
Peake et al., 2017 [35]	Post hoc analysis	Australia	1591	63	60	ED	18.7	Worst values during ED stay	90-day mortality	Early septic shock	Exploration of utility and potential effects of new Sepsis-3 definitions
Quinten et al., 2017 [36]	Prospective, single-center, observational study	The Netherlands	193	60	56	ED	4.1	Initial values measured during admission to ED	In-hospital, 28-day, and 6-month mortality	Suspected or confirmed infection	Comparison of predictive performance of qSOFA, CIS, and PIRO score for ICU admission
Ranzani et al., 2017 [37]	Retrospective, two-center, cohort study	Spain	6874	66	62	ED	6.4	ED arrival	In-hospital mortality	Community-acquired pneumonia	Comparison of predictive performance of SIRS, qSOFA, CRB, mSOFA, and CURB-65 for in-hospital mortality
Seymour et al., 2016 [6]	Retrospective, multicenter, cohort study (in the UPMC validation cohort)	USA	66,522	61	43	ED, ward	2.8	At time of initial suspicion of infection	In-hospital mortality	Suspected infection	Comparison of performance of qSOFA, SIRS, SOFA, and MODS score to predict sepsis
Wang et al., 2016 [38]	Retrospective, single-center, observational study	China	477	73	62	ED	27.4	ED arrival	28-day mortality	Suspected infection	Performance of qSOFA for predicting mortality and ICU admission
Williams et al., 2017 [39]	Retrospective, single-center, observational study	Australia	8871	49	51	ED	8.7	Worst values during ED stay	30-day and 1-year mortality	Suspected infection	Comparison of diagnostic accuracy of SIRS and qSOFA for organ dysfunction and mortality

*Abbreviations: qSOFA* Quick Sequential (Sepsis-related) Organ Failure Assessment, *ED* Emergency department, *ICU* Intensive care unit, *SIRS* Systemic inflammatory response syndrome, *CRB* Confusion, respiratory rate ≥ 30/minute, systolic blood pressure < 90 mmHg or diastolic blood pressure ≤ 60 mmHg, *CRB-65* Confusion, respiratory rate ≥ 30/minute, systolic blood pressure < 90 mmHg or diastolic blood pressure ≤ 60 mmHg, age ≥ 65 years, *CURB-65* Confusion, urea nitrogen, respiratory rate ≥ 30/minute, systolic blood pressure < 90 mmHg or diastolic blood pressure ≤ 60 mmHg, age ≥ 65 years, *NA* Not available, *SK* “Sepsis Kills” program clinical excellence committee, *CIS* Clinical Impression Score, *PIRO* Predisposition, infection, response, organ dysfunction, *mSOFA* Modified Sequential (Sepsis-related) Organ Failure Assessment, *UPMC* University of Pittsburgh Medical Center, *MODS* Multiple organ dysfunction syndrome, *SOFA* Sequential (Sepsis-related) Organ Failure Assessment

### Diagnostic accuracy for in-hospital mortality using positive qSOFA scores and SIRS criteria

**Fig. 2:**
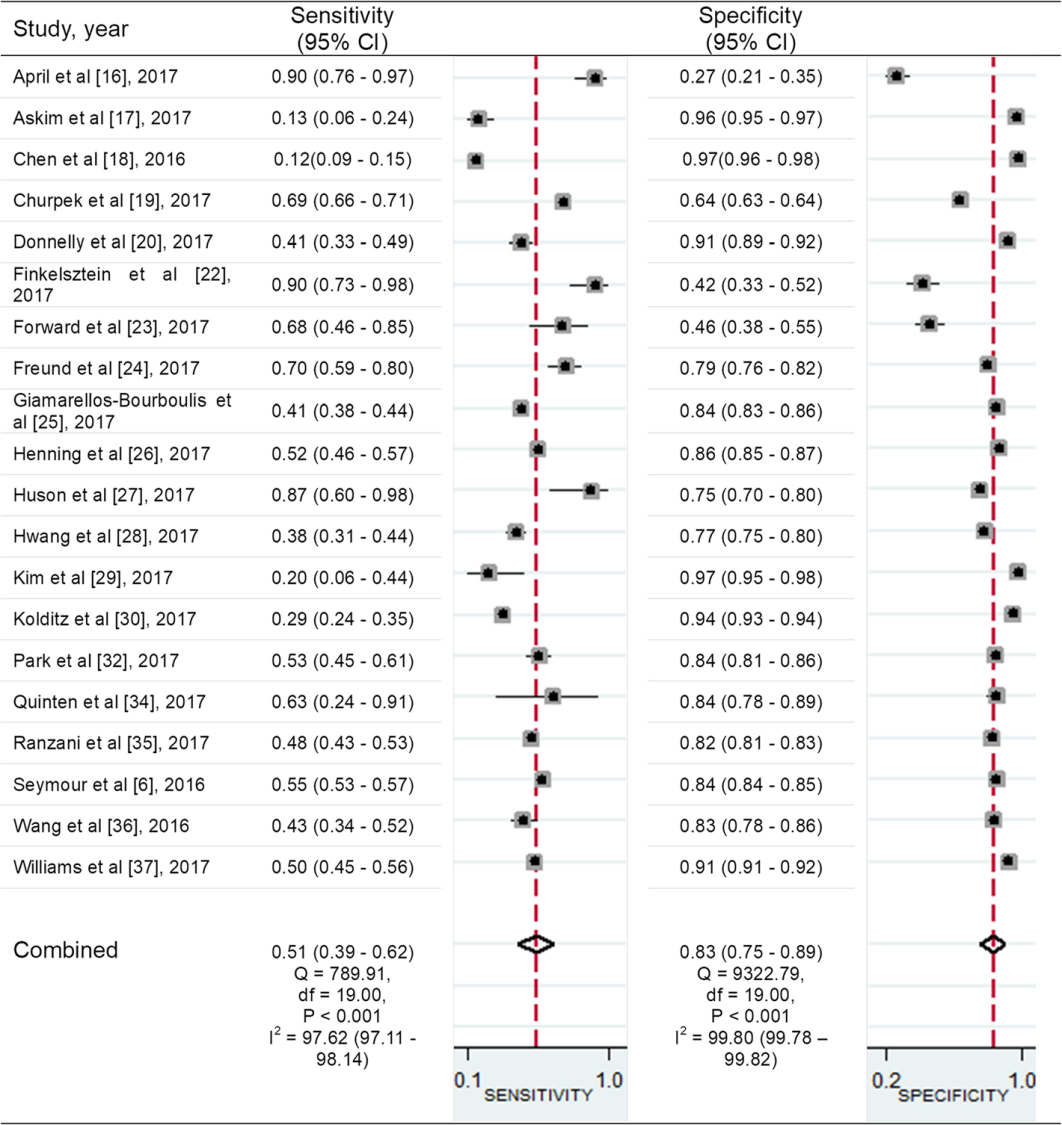
Paired forest plots of sensitivity and specificity of positive quick Sequential (Sepsis-related) Organ Failure Assessment scores in predicting in-hospital mortality in patients with infection outside the intensive care unit

**Fig. 3:**
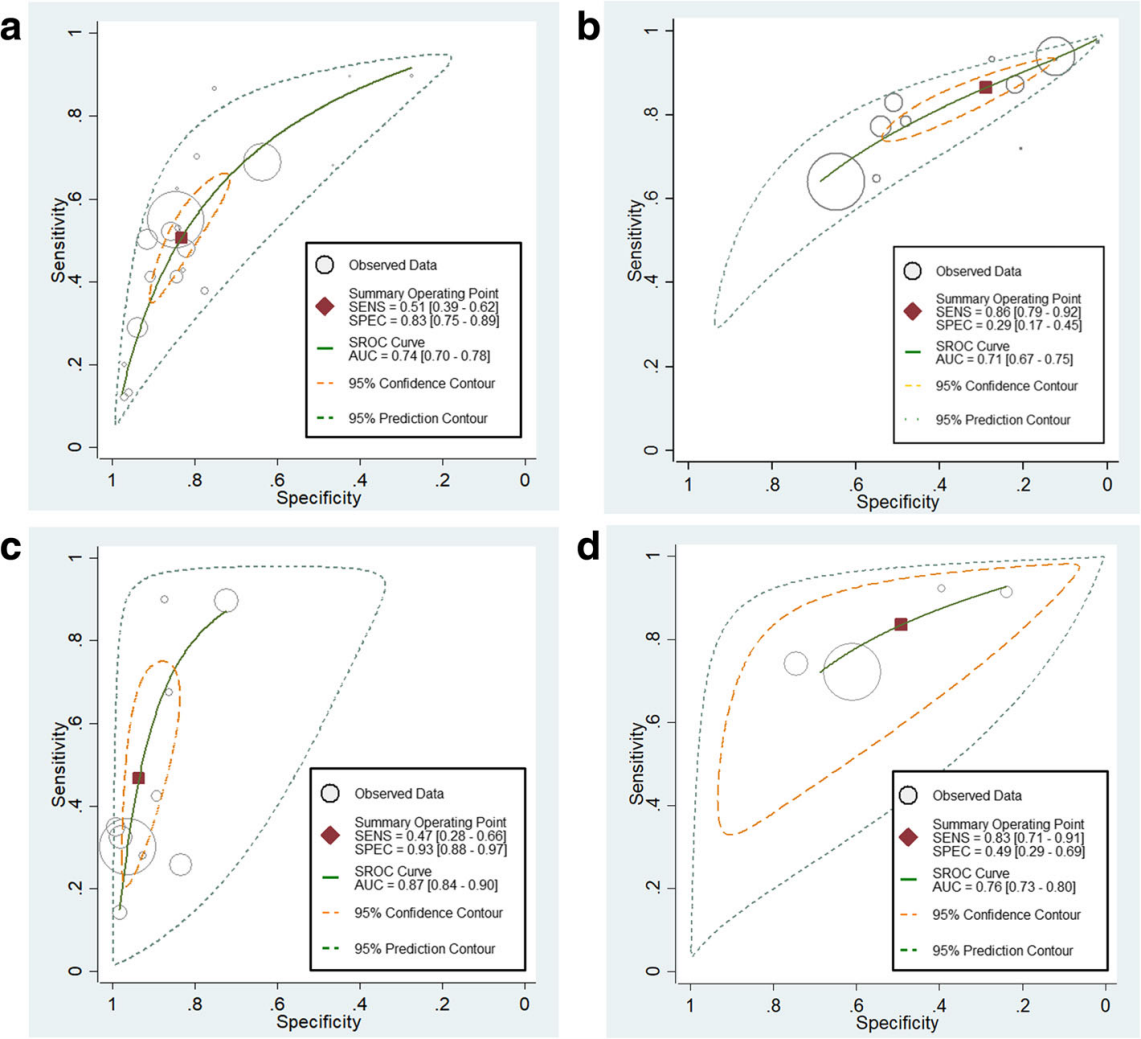
Hierarchical summary ROC (SROC) curves for (**a**) positive quick Sequential (Sepsis-related) Organ Failure Assessment (qSOFA) scores and (**b**) positive systemic inflammatory response syndrome (SIRS) criteria for predicting in-hospital mortality, and for (**c**) positive qSOFA scores and (**d**) positive SIRS criteria for early detection of acute organ dysfunction in patients with infection outside the intensive care unit. *SENS* Sensitivity; *SPEC*, Specificity

In the pooled estimates, patients with positive qSOFA scores and SIRS criteria were associated with in-hospital mortality of 12.9% (3847 of 29,709 patients) and 5.8% (3906 of 67,225 patients), respectively. Using the combined data from the included studies, in Fig. 2 we show the pooled sensitivity and specificity of positive qSOFA scores for in-hospital mortality. The pooled sensitivity and specificity for positive qSOFA scores were 0.51 (95% CI, 0.39–0.62) and 0.83 (95% CI, 0.74–0.89), respectively. The PLR, NLR, and pooled DOR were 3.00 (95% CI, 2.39–3.77), 0.60 (95% CI, 0.50–0.70), and 5.04 (95% CI, 4.09–6.23), respectively. The pooled sensitivity and specificity for positive SIRS criteria were 0.86 (95% CI, 0.79–0.92) and 0.29 (95% CI, 0.17–0.45), respectively. The PLR, NLR, and the pooled DOR were 1.22 (95% CI, 1.06–1.39), 0.46 (95% CI, 0.39–0.56), and 2.59 (95% CI, 1.98–3.38), respectively (*see* Additional file 4). Figure 3a and b show HSROC curves for both tools in predicting in-hospital mortality. The AUC was 0.74 (95% CI, 0.70–0.78) for positive qSOFA scores and 0.71 (95% CI, 0.67–0.75) for positive SIRS criteria. In a comparison of the prognostic performance of the two methods for in-hospital mortality, no significant differences were observed between the AUCs (*P* = 0.816).

In addition, we retrieved data regarding prognostic performance according to the qSOFA score at other cutoff values from three studies [21, 30, 34]. In pooled estimates, when qSOFA was ≥ 1 point, the sensitivity, specificity, and AUC of positive qSOFA scores for in-hospital mortality were 0.93 (95% CI, 0.92–0.94), 0.13 (95% CI, 0.12–0.13), and 0.78 (95% CI, 0.72–0.84), respectively. In the cases with a cutoff value of 3 points, the sensitivity, specificity, and AUC of positive qSOFA scores were 0.17 (95% CI, 0.16–0.19), 0.96 (95% CI, 0.96–0.96), and 0.95 (95% CI, 0.88–1.00), respectively.

### Meta-regression for positive qSOFA scores in predicting in-hospital mortality

Between-study heterogeneity was highly represented in the sensitivities and specificities among the studies (Fig. 2). Table 2 shows the results of univariate meta-regression analyses in identifying potential sources of heterogeneity. For studies in which researchers evaluated the prognostic performance of positive qSOFA scores, study location, overall mortality rate, timing of the qSOFA score measurement, and disease severity were probable sources of heterogeneity. For these four potential sources of heterogeneity, meta-regression analyses using the model weighted by the inverse of the variance revealed that overall mortality ≥ 10% and timing of the qSOFA score measurement were independently associated with between-study heterogeneity (relative diagnostic OR [RDOR], 0.71; 95% CI, 0.53–0.96; *P* = 0.03; and RDOR, 0.59; 95% CI, 0.43–0.81; *P* < 0.01, respectively) (Table 3). For studies with overall mortality ≥ 10% and < 10%, the AUCs were 0.73 (95% CI, 0.67–0.79) and 0.78 (95% CI, 0.76–0.83), respectively. For positive qSOFA scores measured at the time of initial suspicion of infection and the worst values, the AUCs were 0.73 (95% CI, 0.69–0.77) and 0.76 (95% CI, 0.72–0.80), respectively.

**Table 2:** Univariate meta-regression analysis to identify potential sources of heterogeneity in predictive performance of positive quick Sequential (Sepsis-related) Organ Failure Assessment scores for in-hospital mortality outside the intensive care unit

			Sensitivity		Specificity	
Variable	No. of studies	No. of patients	Adjusted (95% CI)	*P* value	Adjusted (95% CI)	*P* value
Study design						
Prospective	4	2759	0.59 (0.32–0.86)	0.68	0.81 (0.64–0.99)	0.30
Retrospective	16	141,778	0.49 (0.36–0.62)		0.84 (0.76–0.91)	
Study location						
USA	6	107,795	0.69 (0.53–0.85)	0.13	0.70 (0.52–0.87)	<0.01
Other countries	14	36,742	0.42 (0.30–0.54)		0.87 (0.81–0.93)	
No. of patients						
≥ 1500	10	5424	0.39 (0.25–0.54)	0.13	0.89 (0.83–0.95)	0.84
< 1500	10	139,113	0.62 (0.47–0.77)		0.74 (0.62–0.86)	
Overall mortality, %						
≥ 10%	10	18,715	0.54 (0.38–0.70)	0.68	0.77 (0.66–0.89)	0.01
< 10%	10	125,822	0.47 (0.31–0.63)		0.88 (0.80–0.95)	
Location of enrollment						
Only ED	11	30,725	0.47 (0.31–0.63)	0.59	0.85 (0.76–0.94)	0.27
Other non-ICU	9	113,812	0.55 (0.38–0.72)		0.81 (0.69–0.92)	
Timing of the qSOFA score measurement						
At time of initial suspicion of infection	13	124,030	0.39 (0.28–0.51)	0.01	0.88 (0.82–0.94)	0.95
Worst values	7	20,507	0.71 (0.57–0.85)		0.72 (0.56–0.87)	
Disease severity						
Suspected or confirmed infection	17	142,776	0.46 (0.34–0.57)	0.18	0.87 (0.82–0.92)	<0.01
Sepsis or septic shock	3	1761	0.74 (0.53–0.96)		0.49 (0.23–0.76)	
Source of infection						
Suspected or confirmed infection	16	126,080	0.58 (0.47–0.69)	0.08	0.79 (0.70–0.87)	0.66
Specific infectious entity (community-acquired pneumonia or neutropenic fever)	4	18,457	0.24 (0.08–0.40)		0.94 (0.89–1.00)	

*Abbreviations: qSOFA* Quick Sequential (Sepsis-related) Organ Failure Assessment, *ED* Emergency department, *ICU* Intensive care unit

**Table 3:** Meta-regression analysis performed using model weighted by the inverse of the variance

Covariates	Coefficient	SE	RDOR (95% CI)^a^	*P* value^b^
Study location	0.04	0.15	1.04 (0.75–1.44)	0.79
Overall mortality ≥ 10%	−0.34	0.14	0.71 (0.53–0.96)	0.03
Timing of qSOFA score measurement	−0.53	0.159	0.59 (0.43–0.81)	<0.01
Disease severity	−0.44	0.26	0.64 (0.37–1.12)	0.11

*qSOFA* Quick Sequential (Sepsis-related) Organ Failure Assessment

^a^The RDOR means the diagnostic OR (DOR) for studies that lacked a particular methodological feature divided by the DOR for studies without the flaw

^b^*P* values from random effects meta-regression using restricted maximum likelihood

### Diagnostic accuracy for acute organ dysfunction using positive qSOFA scores and SIRS criteria

We could retrieve ten data from nine studies regarding the prognostic performance of positive qSOFA scores in predicting acute organ dysfunction [19, 23, 25, 30, 31, 33–35, 39]. Researchers in four studies reported the performance of positive SIRS criteria in predicting acute organ dysfunction [19, 25, 33, 39]. In the pooled estimates, patients with positive qSOFA scores and SIRS criteria were associated with acute organ dysfunction in 82.8% (2433 of 2936 patients) and 36.2% (1830 of 5047 patients), respectively. The pooled sensitivity and specificity of positive qSOFA score for acute organ dysfunction were 0.47 (95% CI, 0.28–0.66) and 0.93 (95% CI, 0.88–0.97), respectively. The PLR, NLR, and pooled DOR were 7.13 (95% CI, 4.42–11.49), 0.57 (95% CI, 0.40–0.81), and 12.49 (95% CI, 6.69–23.31), respectively (*see* Additional file 5). The pooled sensitivity and specificity of positive SIRS criteria were 0.83 (95% CI, 0.71–0.91) and 0.49 (95% CI, 0.29–0.69), respectively. The PLR, NLR, and pooled DOR were 1.64 (95% CI, 1.19–2.26), 0.34 (95% CI, 0.24–0.47), and 4.89 (95% CI, 3.11–7.67), respectively (*see* Additional file 6). Figure 3c and d show HSROC curves for both tools in predicting acute organ dysfunction. The AUC was 0.86 (95% CI, 0.83–0.89) for positive qSOFA score and 0.76 (95% CI, 0.73–0.80) for positive SIRS criteria. In a comparison of the prognostic performance of the two tools for acute organ dysfunction, the AUC for positive qSOFA score was higher than that for positive SIRS criteria (*P* < 0.001).

### Diagnostic accuracy for ICU admission using positive qSOFA scores and SIRS criteria

We could retrieve data from ten studies regarding the prognostic performance of positive qSOFA scores in predicting ICU admission [20, 21, 25, 26, 30–32, 35, 36, 38]. Researchers in three studies reported the performance of positive SIRS criteria in predicting ICU admission [21, 25, 26]. In the pooled estimates, patients with positive qSOFA scores and SIRS criteria were associated with ICU admission of 37.0% (5325 of 14,384 patients) and 24.3% (6741 of 27,759 patients), respectively. The pooled sensitivity and specificity of positive qSOFA score for ICU admission were 0.53 (95% CI, 0.52–0.54) and 0.75 (95% CI, 0.75–0.76), respectively (*see* Additional file 7). The PLR, NLR, and pooled DOR were 2.24 (95% CI, 1.91–2.77), 0.74 (95% CI, 0.67–0.83), and 3.16 (95% CI, 2.42–4.11), respectively. The pooled sensitivity and specificity for positive SIRS criteria were 0.91 (95% CI, 0.90–0.92) and 0.14 (95% CI, 0.13–0.14), respectively. The PLR, NLR, and pooled DOR were 1.11 (95% CI, 0.96–2.26), 0.61 (95% CI, 0.39–0.95), and 1.83 (95% CI, 1.02–3.30), respectively (*see* Additional file 8). Positive qSOFA scores tended to be inferior to positive SIRS criteria in predicting ICU admission, although this was not statistically significant (AUC, 0.63; 95% CI, 0.62–0.64; vs. AUC, 0.78; 95% CI, 0.58–0.98; *P* = 0.121) (*see* Additional file 9).

## Discussion

In the present systematic review and meta-analysis, we analyzed the prognostic performance of positive qSOFA scores for predicting in-hospital mortality in patients with suspected or confirmed infection outside the ICU. We found that positive qSOFA scores had a sensitivity of 0.51 and a specificity of 0.83 for in-hospital mortality as compared with a sensitivity of 0.86 and a specificity of 0.29 for positive SIRS scores. Positive qSOFA scores and SIRS criteria showed similar discrimination for in-hospital mortality (AUC, 0.74 vs. 0.71; *P* = 0.816). Considerable heterogeneity was found in the pooled estimates among the positive qSOFA scores. Using meta-regression analysis, potential sources of heterogeneity were overall mortality and the timing of the qSOFA score measurement. In addition, although the discriminatory capacity of acute organ dysfunction using positive qSOFA score was good, the sensitivity was very low. The sensitivity of positive qSOFA scores in predicting ICU admission was also low.

In two international consensus conferences in 1991 and 2001, sepsis was defined as a suspected source of infection in the setting of SIRS criteria ≥ 2 [8, 9]. For over two decades, the SIRS criteria have been used to be identify sepsis. However, the SIRS criteria have not been useful in differentiating patients with infection outside the ICU from those patients outside the ICU with noninfectious diseases, such as severe trauma, burns, pancreatitis, and ischemia-reperfusion injury [40, 41]. Also, researchers in previous studies reported that 93% of ICU patients and 47% of ward patients who were hospitalized developed positive SIRS at least once during their hospital stay [40, 42]. A large retrospective study showed that a positive SIRS score missed one in eight patients with infection and organ dysfunction [5]. Because of its poor specificity, the SIRS criteria have been regarded as impractical for the screening of sepsis [40, 42].

In the Sepsis-3 guidance, the 2016 SCCM/ESICM proposed the concept of the qSOFA score to predict poor outcomes in patients with suspected infection, and the SIRS criteria were no longer recommended as part of the clinical criteria for sepsis [1]. From the introduction of the new concept, there has been a need to evaluate the prognostic value of qSOFA for predicting outcomes. Several validation studies have followed, and the ability of the qSOFA score to predict in-hospital mortality has been greater than that of the SIRS criteria among patients with suspected infection outside the ICU [6, 28]. We found that patients with positive qSOFA scores were associated with in-hospital mortality of 12.9%, acute organ dysfunction of 82.8%, and ICU admission of 37.0% after the initiation of therapy. In addition, our pooled estimates demonstrated that positive qSOFA scores had high specificity for early risk assessment but poor sensitivity. The qSOFA score would provide great value as a clinical tool to promptly identify patients with infection likely to develop adverse outcomes outside the ICU.

However, we observed wide heterogeneity among the included studies. An important objective of meta-analysis is to investigate the evidence of heterogeneity among studies and to determine whether differences in study design explain the heterogeneity. We found that these findings could be explained partly by potential sources of bias. First, there is the timing of the qSOFA score measurement. Measurement of the qSOFA score at the time of initial suspicion of infection seemed to be easy and clear to apply in clinical practice. However, in pooled estimates, the diagnostic performance of positive qSOFA scores for predicting in-hospital mortality was low at the time of initial suspicion of infection. The use of the worst qSOFA score during the entire stay of the patient enhanced sensitivity. Considering that sepsis is a dynamic, heterogeneous disease, these findings highlight the importance of serial reassessment of the qSOFA score. Second, the severity of infection may affect diagnostic accuracy. When critically ill patients with sepsis or septic shock were included in the meta-analysis, the sensitivity of a positive qSOFA score was enhanced. Otherwise, in pooled estimates targeting patients with common suspected or confirmed infections, the sensitivity of a positive qSOFA score was low. Similarly, pooled estimates for trials that included an overall mortality > 10% increased the sensitivity of a positive qSOFA score. Third, study location may be a factor in heterogeneity. The differences in the results between studies may be explained by differences in the healthcare systems of each country. Specifically, high health care accessibility is likely to be biased toward low disease severity.

Early recognition of sepsis and promptly providing aggressive fluid resuscitation and administration of antimicrobials is crucial to improving outcomes and decreasing sepsis-related mortality [43]. The qSOFA score has an advantage as a simple tool; namely, it has few variables and no necessary laboratory results, and it can be assessed repeatedly over time. However, the qSOFA score reflects only some of the variables in the new sepsis definition. In our pooled estimates, its low sensitivity, which may lead to delays in initiation of adequate management for some patients, has resulted in concerns about its role as a bedside tool outside the ICU [23]. To facilitate the early recognition of patients at higher risk for poor outcomes, some specificity of the qSOFA score would need to be sacrificed to increase sensitivity [23]. Therefore, its ability to predict mortality may be enhanced when combined with other clinical factors that are correlated with higher risk of death and acute organ dysfunction, such as age, nursing home residence, arterial pH, and lactate and end-tidal carbon dioxide concentrations [23, 27, 28]. In addition, a recent retrospective study reported that the diagnostic accuracy was highest in predicting acute organ dysfunction in the ED when the cutoff qSOFA score was ≥ 1 point [34]. However, in our pooled estimates of the qSOFA score ≥ 1 point for in-hospital mortality, although the pooled sensitivity increased, specificity largely decreased. The findings from a recent observational cohort study were consistent with our results [21]. Also, the qSOFA score ≥ 1 point had diagnostic accuracy similar to positive SIRS criteria for in-hospital mortality or ICU transfer, which suggested that this lower cutoff could be used to enhance the sensitivity of the qSOFA score [21]. This study also reported that other early warning scores such as the Modified Early Warning Score and the National Early Warning Score were more accurate than the qSOFA score for predicting adverse outcomes outside the ICU, and, owing to the costs, its authors did not recommend changing from these other early warning scores to the qSOFA score [21].

The strengths of our study are that we (1) followed a standard protocol using a comprehensive search strategy, (2) applied a bivariate random effects model and HSROC analyses to the data, and (3) identified potential sources of bias by adding covariates to the bivariate model for meta-regression. Our findings could be useful for physicians implementing qSOFA outside the ICU. Meanwhile, there are some study limitations. First, there was significant heterogeneity among the included studies, although large heterogeneities are commonly seen in systematic reviews of diagnostic test accuracy studies [44]. Second, although we performed meta-regression analysis to determine which studies contributed to the observed heterogeneity, the results of this analysis should be interpreted with caution owing to limited statistical power. Third, we found only four and three studies using positive SIRS criteria for acute organ dysfunction and ICU admission, respectively. Although our results reveal that the discriminatory capacity for acute organ dysfunction of positive qSOFA scores was higher than positive SIRS criteria, limited data did not allow us to draw a robust conclusion. Finally, although the qSOFA score was a bedside criterion targeting patients with suspected infection, a considerable number of patients who were included in this meta-analysis had already-confirmed infections. This could lead to an overestimation of the predictive ability of positive qSOFA scores as well as positive SIRS criteria.

## Conclusions

We found that positive qSOFA scores had high specificity but poor sensitivity for predicting in-hospital mortality, acute organ dysfunction, and ICU admission in patients with infection outside the ICU. Therefore, a positive qSOFA score seemed to be limited in the early identification of poor outcomes in these patients in routine clinical practice. Meanwhile, positive SIRS criteria were found to be too sensitive and insufficiently specific to predict in-hospital mortality. In the present study, between-study heterogeneity was highly represented, and an overall mortality rate ≥ 10% and timing of qSOFA score measurement could be significant factors in this heterogeneity. Our findings indicate that the development of enhanced or modified bedside tools may be necessary.

## Additional files

Additional file 1: Study protocol, search strategies, and quality assessment. (PDF 115 kb)

Additional file 2: Summary for risk of bias of included studies and risk of bias graph for the included studies. (PDF 32 kb)

Additional file 3: Funnel plot for publication bias assessment of studies for (**a**) positive qSOFA score and (**b**) positive SIRS criteria score for the prediction of in-hospital mortality. (PDF 97 kb)

Additional file 4: Paired forest plots of sensitivity and specificity of positive SIRS criteria in predicting in-hospital mortality of infected patients outside the intensive care unit. (PDF 268 kb)

Additional file 5: Paired forest plots of sensitivity and specificity of positive qSOFA score in identifying organ dysfunction in infected patients outside the intensive care unit. (PDF 263 kb)

Additional file 6: Paired forest plots of sensitivity and specificity of positive SIRS criteria score in identifying organ dysfunction in infected patients outside the intensive care unit. (PDF 83 kb)

Additional file 7: Paired forest plots of sensitivity and specificity of positive qSOFA score in identifying ICU admission in infected patients outside the intensive care unit. (PDF 170 kb)

Additional file 8: Paired forest plots of sensitivity and specificity of positive SIRS criteria in identifying ICU admission in infected patients outside the intensive care unit. (PDF 107 kb)

Additional file 9: Summary receiver operating characteristic curves for (**a**) positive qSOFA score and (**b**) positive SIRS criteria for predicting ICU admission in infected patients outside the intensive care unit. (PDF 77 kb)

## Abbreviations

CIS: Clinical Impression Score
CRB: Confusion, respiratory rate ≥ 30/minute, systolic blood pressure < 90 mmHg or diastolic blood pressure ≤ 60 mmHg
CRB-65: Confusion, respiratory rate ≥ 30/minute, systolic blood pressure < 90 mmHg or diastolic blood pressure ≤ 60 mmHg, age ≥ 65 years
CURB-65: Confusion, urea nitrogen, respiratory rate ≥ 30/minute, systolic blood pressure < 90 mmHg or diastolic blood pressure ≤ 60 mmHg, age ≥ 65 years
DOR: Diagnostic OR
ED: Emergency department
HSROC: Hierarchical summary ROC
ICU: Intensive care unit
MODS: Multiple organ dysfunction syndrome
mSOFA: Modified Sequential (Sepsis-related) Organ Failure Assessment
NA: Not available
NLR: Negative likelihood ratio
PIRO: Predisposition, infection, response, organ dysfunction model
PLR: Positive likelihood ratio
qSOFA: Quick Sequential (Sepsis-related) Organ Failure Assessment
QUADAS-2: Quality Assessment of Diagnostic Accuracy Studies 2 tool
RDOR: Relative diagnostic OR
SCCM/ESICM: Society of Critical Care Medicine/European Society of Intensive Care Medicine
SENS: Sensitivity
Sepsis-3: SCCM/ESICM Third International Consensus Definitions for Sepsis and Septic Shock
SIRS: Systemic inflammatory response syndrome
SK: “Sepsis Kills” program clinical excellence committee
SOFA: Sequential (Sepsis-related) Organ Failure Assessment
SPEC: Specificity
SROC: Summary ROC
UPMC: University of Pittsburgh Medical Center
