# Supplementary material for: Performance of the quick Sequential (sepsis-related) Organ Failure Assessment score as a prognostic tool in infected patients outside the intensive care unit: a systematic review and meta-analysis
Source: Crit Care. 2018 Feb 6;22:28. doi: 10.1186/s13054-018-1952-x (PMC5802050; doi:10.1186/s13054-018-1952-x)
Supplement: Supplementary file 1 — Study protocol, search strategies, and quality assessment. (PDF 115 kb) [file 13054_2018_1952_MOESM1_ESM.pdf]

Additional File 1. Study Protocol, Search Strategies, and Quality assessment

Study protocol for systematic review and meta-analysis of the performance of the quick sequential organ failure assessment score as a prognostic tool for mortality and organ failure in infected patients outside the intensive care unit

Objective

1. Using a meta-analysis strategy to evaluate the prognostic value of positive the Quick Sequential [Sepsis-related] Organ Function Assessment (qSOFA) score for early identification of in-hospital mortality and organ dysfunction in patients with suspected or confirmed infection outside the intensive care unit (ICU).

2. To compare the discriminatory capacity between a positive qSOFA score and positive Systemic Inflammatory Response Syndrome (SIRS) criteria for predicting in-hospital mortality.

Inclusion Criteria

Study type

- We will include studies assessing qSOFA as an early, prognostic tool to identify in-hospital mortality and organ dysfunction in infected patients outside the ICU.

Participants

- Eligible studies should include patients with suspected or confirmed infection outside the ICU.
- Eligible studies should include patients with qSOFA score.
- A positive qSOFA score was defined as meeting two or more of the following criteria: respiratory rate ≥22 breaths/min, systolic blood pressure <100 mmHg, or altered mentation.
- A positive SIRS criteria score was defined as meeting two or more of the following criteria: respiratory rate ≥20 breaths/min, temperature ≥38℃ or <36℃, heart rate ≥90 beats/min, and white blood cell count ≥12,000/mm^3 or <4,000/mm^3, or ≥10% bands.

Outcome measures

- Eligible studies should report cases of true positive, false positive, false negative, and true negative results in absolute numbers, or these data can be derived from the presented results.

Publication type

- Full-length articles or letters in peer-reviewed English-language journals were eligible.
- This research was limited to human studies.
- Studies published solely in abstract form were excluded because the methods and results could not be completely analyzed.

Search strategies

MEDLINE

1. (sepsis [tiab] OR sepsis-3 [tiab] OR septic shock [tiab]) OR “sepsis” [Mesh:NoExp] 115,529

2. validation [ti] OR mortality [ti] 151,761

3. 1 AND 2 3,657

4. quick sepsis-related organ failure assessment [tiab] OR quick sequential organ failure assessment [tiab] OR quick sequential organ dysfunction [tiab] OR qSOFA [tiab] 66

5. 3 OR 4 3,709

EMBASE

1. sepsis:ab,ti OR sepsis-3:ab,ti OR septic shock:ab,ti 4029 OR ‘sepsis’/de OR ‘sepsis’/exp 216,682

2. validation:ti OR mortality:ti 197,898

3. 1 and 2 6,691

4. quick sepsis-related organ failure assessment:ab,ti OR quick sequential organ failure assessment:ab,ti OR quick sequential organ dysfunction:ab,ti OR qSOFA:ab,ti 85

5. 3 OR 4 6,758

COCHRANE CENTRAL REGISTER

1. validation OR mortality:ti,ab,kw 51,861

2. MeSH descriptor: [sepsis] explode all trees 3,576

3. 1-2/AND 867

4. quick sepsis-related organ failure assessment OR quick sequential organ failure assessment OR quick sequential organ dysfunction OR qSOFA:ti,ab,kw 26

5. 3 OR 4 893

Quality Assessment

We used the Quality Assessment of Diagnostic Accuracy Studies (QUADAS)-2 tool to assess the risk of bias in diagnostic test accuracy. The QUADAS-2 tool consists of four key domains that discuss patient selection, index test, reference standard, and the flow/timing of patients. The Additional file 2 summarizes the seven items selected to assess risk of bias and applicability. The answer to each item was “yes,” “no” or “unclear” (“yes” indicates a low risk of bias, “no” indicates a high risk of bias, and “unclear” indicates an unclear risk of bias). If a study was judged as “low” on all domains relating to bias or applicability, then it was assigned an overall judgment of “low risk of bias” or “low concern regarding applicability.” If a study was judged “high” or “unclear” in one or more domains, then it may have been judged as “at risk of bias” or “concerns regarding applicability.” Discrepancies were resolved by consensus between two of the authors (J-U.S. and J.L.).
