# Supplementary material for: Performance of the quick Sequential (sepsis-related) Organ Failure Assessment score as a prognostic tool in infected patients outside the intensive care unit: a systematic review and meta-analysis
Source: Crit Care. 2018 Feb 6;22:28. doi: 10.1186/s13054-018-1952-x (PMC5802050; doi:10.1186/s13054-018-1952-x)
Supplement: Supplementary file 2 — Summary for risk of bias of included studies and risk of bias graph for the included studies. (PDF 32 kb) [file 13054_2018_1952_MOESM2_ESM.pdf]

Additional File 2. Summary for risk of bias of included studies and Risk of bias graph for the included studies

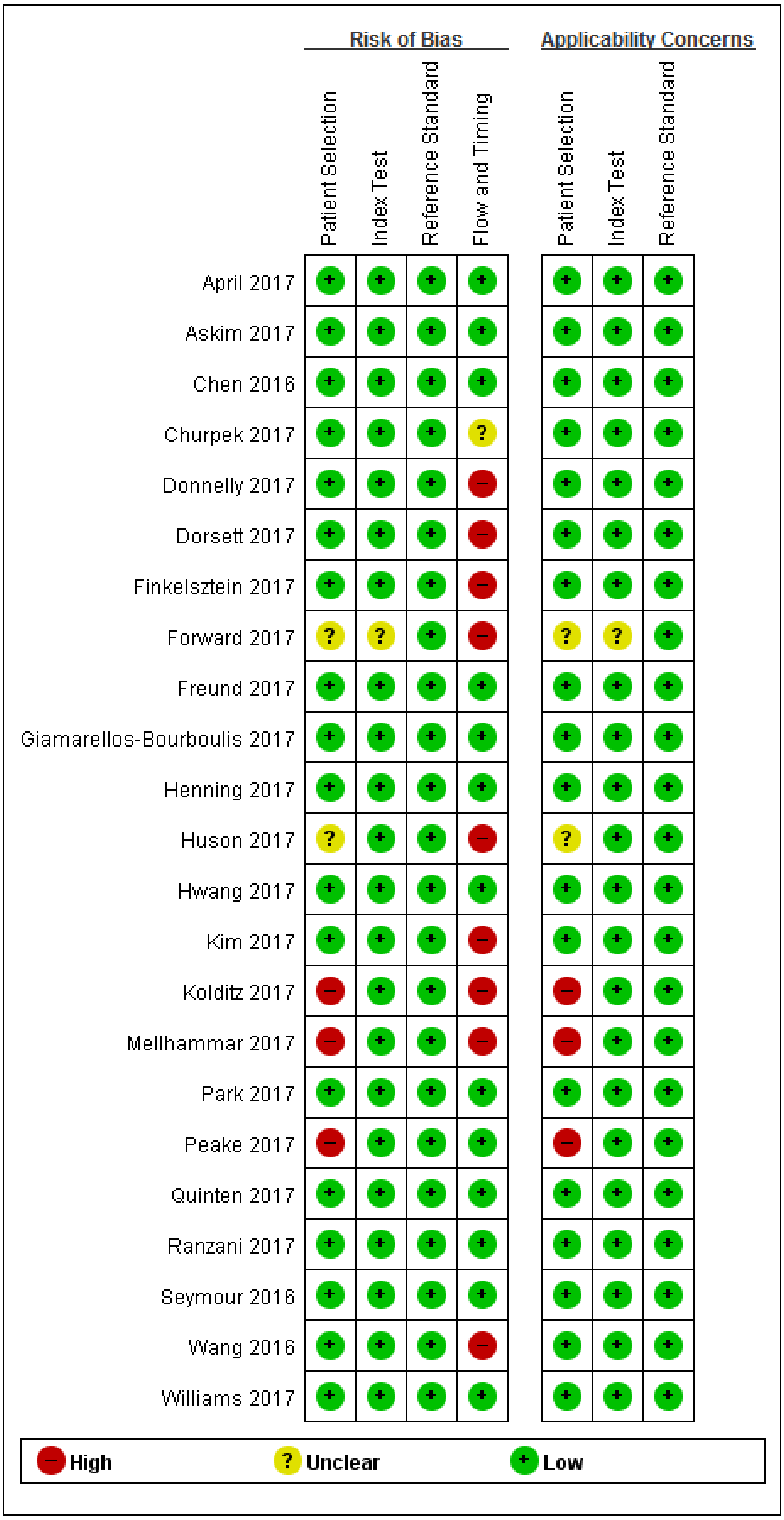

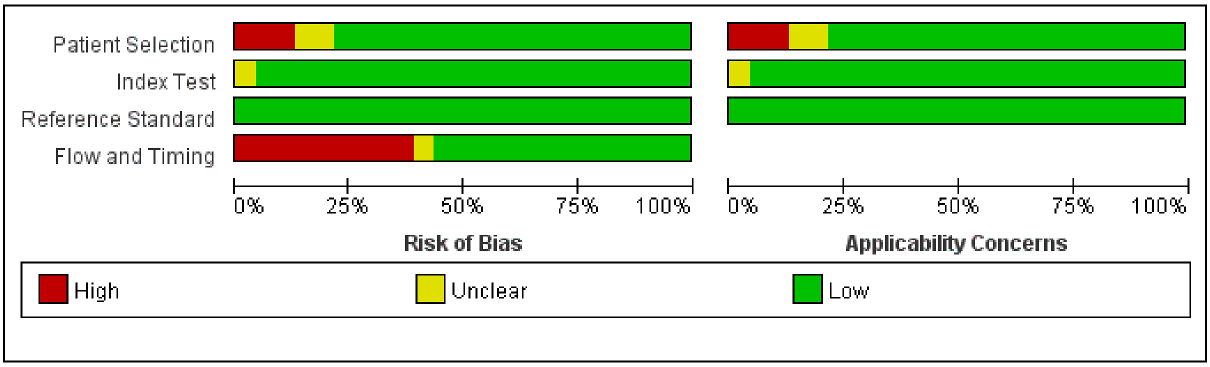

Patient Selection
Index Test
Reference Standard
Flow and Timing
0%
25%
50%
75%
100%
Risk of Bias
Applicability Concerns
High
Unclear
Low
