# Supplementary material for: Performance of the quick Sequential (sepsis-related) Organ Failure Assessment score as a prognostic tool in infected patients outside the intensive care unit: a systematic review and meta-analysis
Source: Crit Care. 2018 Feb 6;22:28. doi: 10.1186/s13054-018-1952-x (PMC5802050; doi:10.1186/s13054-018-1952-x)

Additional File 3. Funnel plot for publication bias assessment of studies for (A) positive qSOFA score and (B) positive SIRS criteria score for the prediction of in-hospital mortality

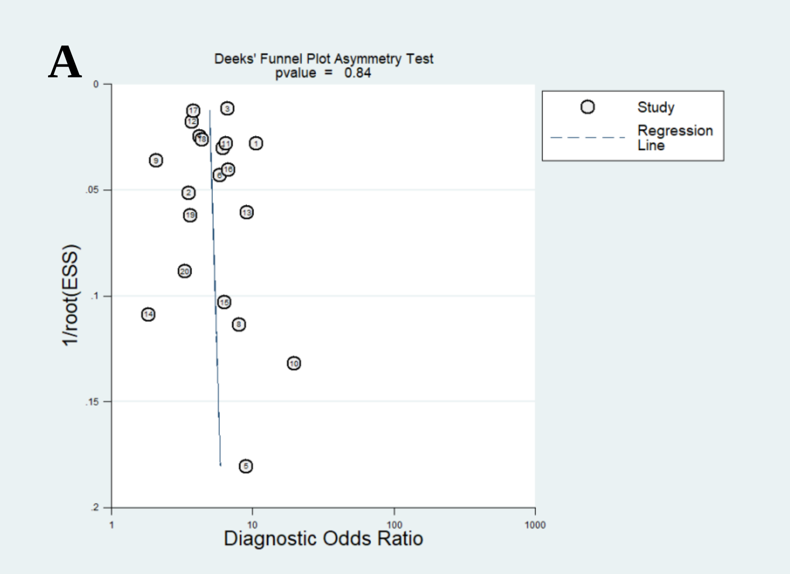

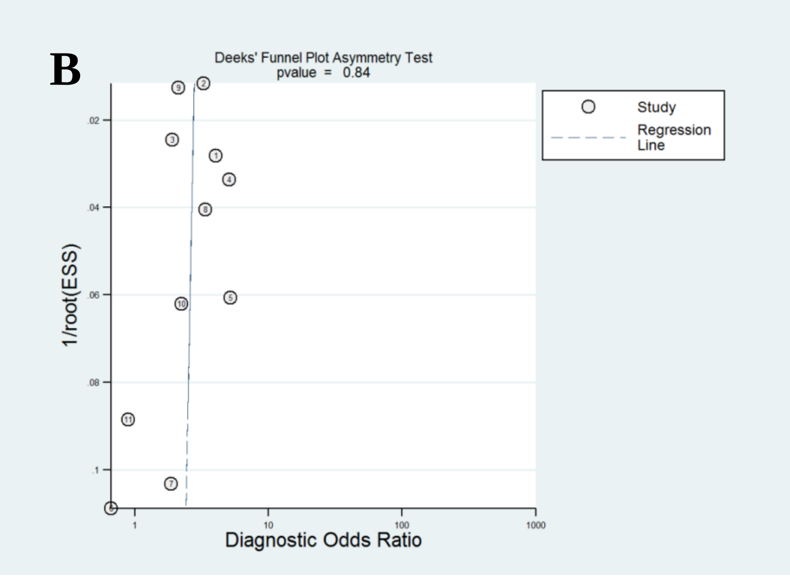

Supplement: Supplementary file 3 — Funnel plot for publication bias assessment of studies for (a) positive qSOFA score and (b) positive SIRS criteria score for the prediction of in-hospital mortality. (PDF 97 kb) [file 13054_2018_1952_MOESM3_ESM.pdf]
