# Supplementary material for: Performance of the quick Sequential (sepsis-related) Organ Failure Assessment score as a prognostic tool in infected patients outside the intensive care unit: a systematic review and meta-analysis
Source: Crit Care. 2018 Feb 6;22:28. doi: 10.1186/s13054-018-1952-x (PMC5802050; doi:10.1186/s13054-018-1952-x)

Additional File 4. Paired forest plots of sensitivity and specificity of positive SIRS criteria in predicting in-hospital mortality of infected patients outside the intensive care unit

Study, year	Sensitivity (95% CI)	Specificity (95% CI)
April et al [16], 2017	0.97 (0.87 – 1.00)	0.02 (0.01 - 0.06)
Askim et al [17], 2017	0.65 (0.52 - 0.76)	0.55 (0.52 - 0.58)
Churpek et al [19], 2017	0.94 (0.93 - 0.95)	0.12 (0.12 - 0.13)
Donnelly et al [20], 2017	0.79 (0.71 - 0.85)	0.48 (0.46 - 0.50)
Finkelsztein et al [22], 2017	0.93 (0.77 - 0.99)	0.12 (0.07 - 0.19)
Forward et al [23], 2017	0.72 (0.51 - 0.88)	0.21 (0.14 - 0.28)
Freund et al [24], 2017	0.93 (0.85 - 0.98)	0.27 (0.24 - 0.31)
Henning et al [26], 2017	0.83 (0.77 - 0.88)	0.51 (0.50 - 0.52)
Ranzani et al [35], 2017	0.87 (0.84 - 0.90)	0.22 (0.21 - 0.23)
Seymour et al [6], 2016	0.64 (0.62 - 0.66)	0.65 (0.64 - 0.65)
Williams et al [37], 2017	0.77 (0.72 - 0.82)	0.54 (0.53 - 0.55)
Combined	0.86 (0.79 - 0.92) Q = 587.52, df = 10.00, P < 0.001 I^2 = 98.30 (97.84 – 98.75)	0.29 (0.17 - 0.45) Q = 38934.63, df = 10.00, P < 0.001 I^2 = 99.97 (99.97 – 99.98)

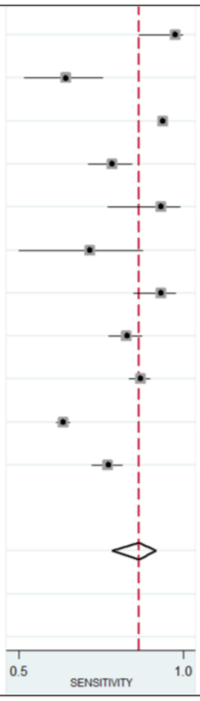

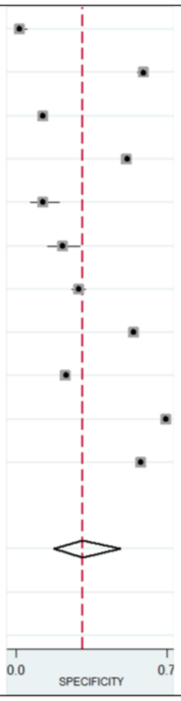

Supplement: Supplementary file 4 — Paired forest plots of sensitivity and specificity of positive SIRS criteria in predicting in-hospital mortality of infected patients outside the intensive care unit. (PDF 268 kb) [file 13054_2018_1952_MOESM4_ESM.pdf]
