# Supplementary material for: Performance of the quick Sequential (sepsis-related) Organ Failure Assessment score as a prognostic tool in infected patients outside the intensive care unit: a systematic review and meta-analysis
Source: Crit Care. 2018 Feb 6;22:28. doi: 10.1186/s13054-018-1952-x (PMC5802050; doi:10.1186/s13054-018-1952-x)
Supplement: Supplementary file 5 — Paired forest plots of sensitivity and specificity of positive qSOFA score in identifying organ dysfunction in infected patients outside the intensive care unit. (PDF 263 kb) [file 13054_2018_1952_MOESM5_ESM.pdf]

Additional File 5. Paired forest plots of sensitivity and specificity of positive qSOFA score in identifying organ dysfunction in infected patients outside the intensive care unit

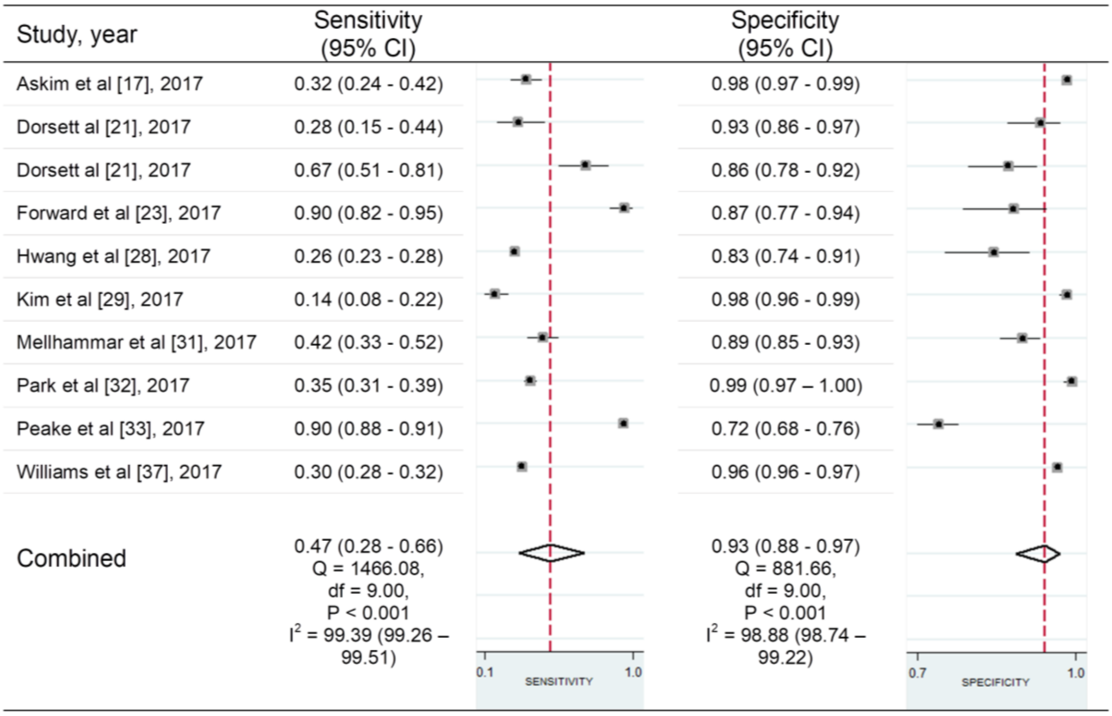

Study, year	Sensitivity (95% CI)	Specificity (95% CI)
Askim et al [17], 2017	0.32 (0.24 - 0.42)	0.98 (0.97 - 0.99)
Dorsett al [21], 2017	0.28 (0.15 - 0.44)	0.93 (0.86 - 0.97)
Dorsett al [21], 2017	0.67 (0.51 - 0.81)	0.86 (0.78 - 0.92)
Forward et al [23], 2017	0.90 (0.82 - 0.95)	0.87 (0.77 - 0.94)
Hwang et al [28], 2017	0.26 (0.23 - 0.28)	0.83 (0.74 - 0.91)
Kim et al [29], 2017	0.14 (0.08 - 0.22)	0.98 (0.96 - 0.99)
Mellhammar et al [31], 2017	0.42 (0.33 - 0.52)	0.89 (0.85 - 0.93)
Park et al [32], 2017	0.35 (0.31 - 0.39)	0.99 (0.97 – 1.00)
Peake et al [33], 2017	0.90 (0.88 - 0.91)	0.72 (0.68 - 0.76)
Williams et al [37], 2017	0.30 (0.28 - 0.32)	0.96 (0.96 - 0.97)
Combined	0.47 (0.28 - 0.66) Q = 1466.08, df = 9.00, P < 0.001 I^2 = 99.39 (99.26 – 99.51)	0.93 (0.88 - 0.97) Q = 881.66, df = 9.00, P < 0.001 I^2 = 98.88 (98.74 – 99.22)
