# Supplementary material for: Performance of the quick Sequential (sepsis-related) Organ Failure Assessment score as a prognostic tool in infected patients outside the intensive care unit: a systematic review and meta-analysis
Source: Crit Care. 2018 Feb 6;22:28. doi: 10.1186/s13054-018-1952-x (PMC5802050; doi:10.1186/s13054-018-1952-x)

Additional File 6. Paired forest plots of sensitivity and specificity of positive SIRS criteria score in identifying organ dysfunction in infected patients outside the intensive care unit

Study, year	Sensitivity (95% CI)	Specificity (95% CI)
Askim et al [17], 2017	0.74 (0.65 - 0.82)	0.74 (0.72 - 0.77)
Forward et al [23], 2017	0.92 (0.85 - 0.97)	0.39 (0.28 - 0.52)
Mellhammar et al [31], 2017	0.91 (0.85 - 0.96)	0.24 (0.19 - 0.30)
Williams et al [37], 2017	0.72 (0.70 - 0.74)	0.61 (0.60 - 0.62)
Combined	0.83 (0.71 - 0.91) Q = 37.53, df = 3.00, P < 0.001 I^2 = 92.01 (85.85 – 98.16)	0.49 (0.29 - 0.69) Q = 287.58, df = 3.00, P < 0.001 I^2 = 98.96 (98.53 – 99.39)

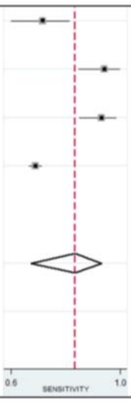

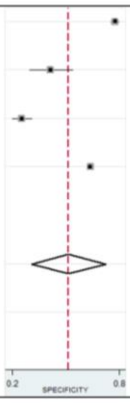

Supplement: Supplementary file 6 — Paired forest plots of sensitivity and specificity of positive SIRS criteria score in identifying organ dysfunction in infected patients outside the intensive care unit. (PDF 83 kb) [file 13054_2018_1952_MOESM6_ESM.pdf]
