# Supplementary material for: Performance of the quick Sequential (sepsis-related) Organ Failure Assessment score as a prognostic tool in infected patients outside the intensive care unit: a systematic review and meta-analysis
Source: Crit Care. 2018 Feb 6;22:28. doi: 10.1186/s13054-018-1952-x (PMC5802050; doi:10.1186/s13054-018-1952-x)

Additional File 7. Paired forest plots of sensitivity and specificity of positive qSOFA score in identifying ICU admission in infected patients outside the intensive care unit

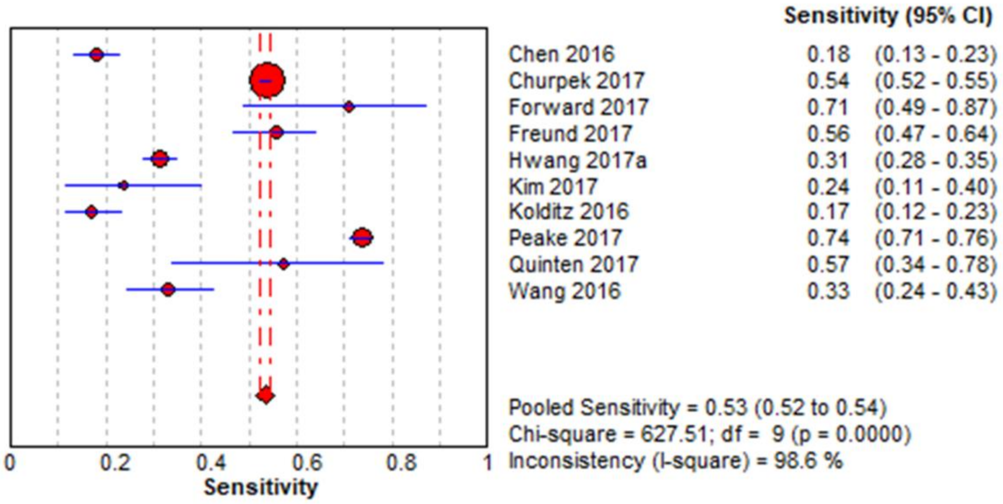

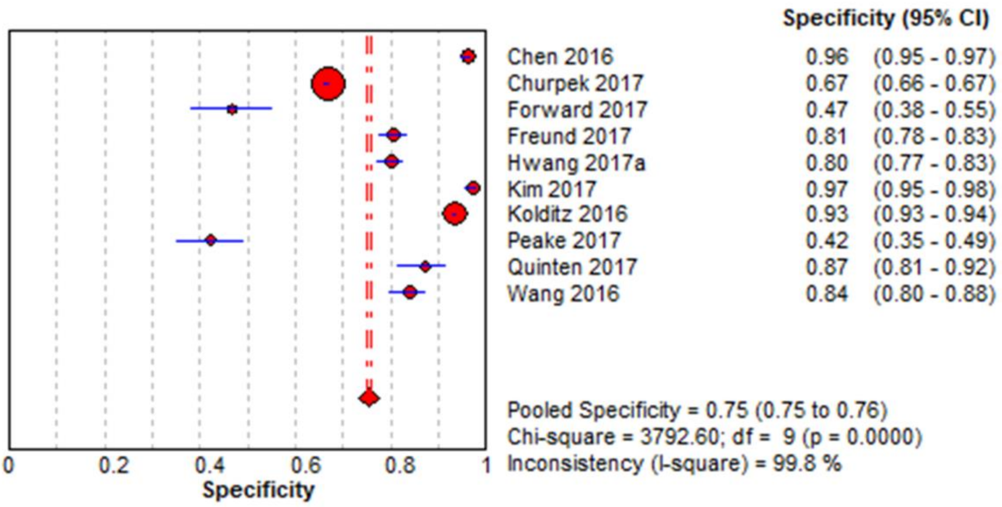

Supplement: Supplementary file 7 — Paired forest plots of sensitivity and specificity of positive qSOFA score in identifying ICU admission in infected patients outside the intensive care unit. (PDF 170 kb) [file 13054_2018_1952_MOESM7_ESM.pdf]
