# Supplementary material for: Performance of the quick Sequential (sepsis-related) Organ Failure Assessment score as a prognostic tool in infected patients outside the intensive care unit: a systematic review and meta-analysis
Source: Crit Care. 2018 Feb 6;22:28. doi: 10.1186/s13054-018-1952-x (PMC5802050; doi:10.1186/s13054-018-1952-x)

Additional File 9. Summary receiver operating characteristic curves for (A) positive qSOFA score and (B) positive SIRS criteria for predicting ICU admission in infected patients outside the intensive care unit.

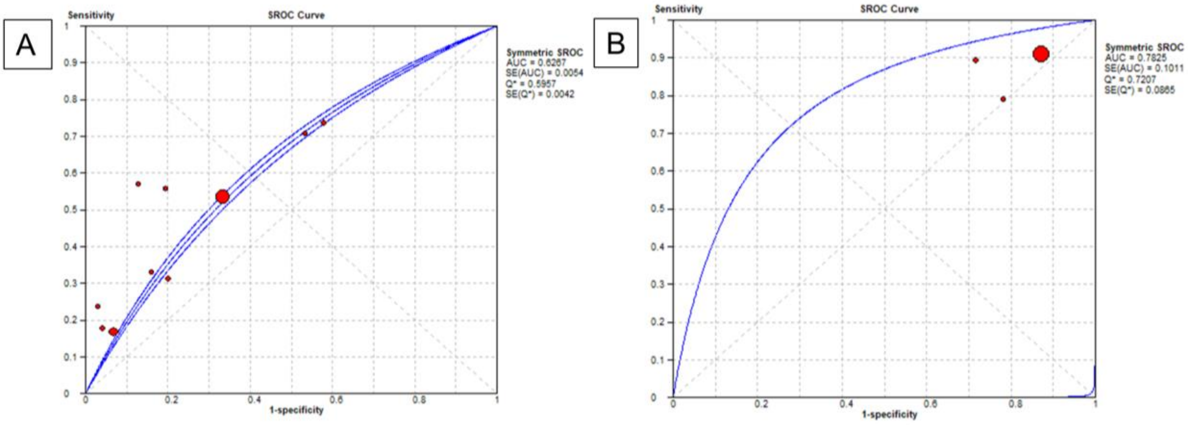

Supplement: Supplementary file 9 — Summary receiver operating characteristic curves for (a) positive qSOFA score and (b) positive SIRS criteria for predicting ICU admission in infected patients outside the intensive care unit. (PDF 77 kb) [file 13054_2018_1952_MOESM9_ESM.pdf]
